# Self‐Healable Fluorinated Copolymers Governed by Dipolar Interactions

**DOI:** 10.1002/advs.202101399

**Published:** 2021-07-06

**Authors:** Siyang Wang, Marek W. Urban

**Affiliations:** ^1^ Department of Materials Science and Engineering Clemson University Clemson SC 29634 USA

**Keywords:** self‐healing polymers, fluoropolymers, van der Waals dipolar interactions

## Abstract

Although dipolar forces between copolymer chains are relatively weak, they result in ubiquitous inter‐ and/or intramolecular interactions which are particularly critical in achieving the mechanical integrity of polymeric materials. In this study, a route is developed to obtain self‐healable properties in thermoplastic copolymers that rely on noncovalent dipolar interactions present in essentially all macromolecules and particularly fluorine‐containing copolymers. The combination of dipolar interactions between C─F and C═O bonds as well as CH_2_/CH_3_ entities facilitates self‐healing without external intervention. The presence of dipole‐dipole, dipole‐induced dipole, and induced‐dipole induced dipole interactions leads to a viscoelastic response that controls macroscopic autonomous multicycle self‐healing of fluorinated copolymers under ambient conditions. Energetically favorable dipolar forces attributed to monomer sequence and monomer molar ratios induces desirable copolymer tacticities, enabling entropic energy recovery stored during mechanical damage. The use of dipolar forces instead of chemical or physical modifications not only eliminates additional alternations enabling multiple damage‐repair cycles but also provides further opportunity for designing self‐healable commodity thermoplastics. These materials may offer numerous applications, ranging from the use in electronics, ion batteries, H_2_ fuel dispense hoses to self‐healable pet toys, packaging, paints and coatings, and many others.

## Graphical abstract

Fluorine‐containing acrylic‐based copolymers that rely on noncovalent dipolar interactions between CH_2_/CH_3_ entities as well as C─F and C═O bonds result in a viscoelastic response leading to macroscopic autonomous self‐healing. The presence of favorable dipolar forces is attributed to monomer sequences and monomer molar ratios facilitating desirable copolymer tacticity, enabling entropic energy storage, and recovery during multiple damage‐repair cycles.

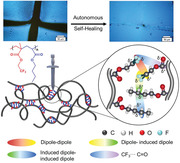

## 1 Introduction

Due to the presence of most electronegative F atom making the C─F bond highly polar fluorinated polymers exhibit many unique properties. Despite the polar nature of the C─F bond, perfluorinated linear fluorinated alkanes exhibit practically no permanent dipole due to the symmetric distribution of C─F bonds on both sides of the polymer backbone. Fluorine atoms are often viewed as a protective sheath surrounding the C─C copolymer backbone and the nonpolar nature of this class of fluorinated polymers is responsible for an array of attractive properties ranging from chemical inertness to thermal stability, or low coefficients of friction, to name just a few.^[^ ^1^ ^]^ However, if CF_3_ groups are asymmetrically positioned or unevenly distributed along the polymer backbone, C─F bonds will display permanent dipole, which is often responsible for the ability to control self‐assembly of fluoropolymers or intracellular trafficking in biological systems. Owing to the fluorophilic effect, fluorinated oligomers are unique candidates in protein and gene delivery systems as well as efficient drugs. The question though is how the high polarity of asymmetrically positioned CF_3_ groups along the polymer backbone with the permanent dipole will impact interactions with the neighboring chains of the same or similar topologies in amorphous fluorinated copolymers. Although it is anticipated that dipole‐dipole (D‐D) and dipole‐inducted dipole (D‐ID) or induced dipole‐induced dipole (ID‐ID) will dominate inter‐ and intrachain interactions, particularly that the CF_3_ groups exhibit a hemispherical volume of 42.6 Å^3^ compared to CH_3_ with 16.8 Å^3^,^[^ ^2^ ^]^ the question is what will be the response of the neighboring chains if these interactions are disrupted by external mechanical separation.

## 2 Results and Discussion

Taking advantage of dipolar interactions between neighboring chains^[^ ^3^ ^]^ we developed a new family of fluorinated acrylic‐based copolymers with self‐healing properties composed of repeating units containing side‐groups terminated with CH_2_/CH_3_ and/or CF_3_ moieties. Of particular interest are H⋅⋅⋅H, F⋅⋅⋅F, and H⋅⋅⋅F through space inter‐ and intrachain interactions, which are sensitive to the reorientation energy barrier during damage‐repair cycle. When external forces are applied to such thermoplastics resulting in conformational changes, these nuclei spin‐spin “communications” will be impacted due to through‐space interactions.

**Figure 1:**
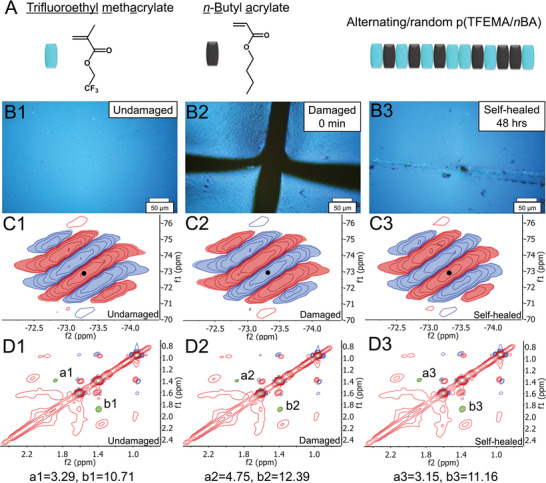
Chemical structure of 2,2,2‐trifluoroethyl methacrylate (TFEMA) and *n*‐butyl acrylate (*n*BA) as well as p(TFEMA/*n*BA) copolymers. A) Optical images of p(TFEMA/*n*BA) copolymer films (50/50 TFEMA/*n*BA monomer molar ratio): B1) undamaged, B2) damaged—cut size: ≈50 µm, and B3) self‐healed. Corresponding 2D ^19^F NOESY NMR spectra of C1) undamaged, C2) damaged, and C3) self‐healed; the solid circle at the center of the – 73.2 ppm resonance marks the resonance due to F nucleus exchange with H and/or other F magnetic environments. 2D^1^H NOESY NMR spectra of D1) undamaged, D2) damaged, and D3) self‐healed copolymer with symmetrical (1.88, 1.39)/(1.39, 1.88) resonances (zoomed in from 0.7 to 2.5 ppm). Movie (Movie S1, Supporting Information) illustrates damage‐repair process.

To identify the nature of inter‐ and intramolecular interactions we copolymerized 2,2,2‐trifluoroethyl methacrylate (TFEMA) and *n*‐butyl acrylate (*n*BA) monomers using radical polymerization (**Figure** **1**A and the Supporting Information) that resulted in poly(2,2,2 trifluoroethyl methacrylate/*n*‐butyl acrylate) [p(TFEMA/*n*BA)] copolymers. By varying TFEMA/*n*BA monomer molar ratios from 30/70 to 70/30 self‐healing was examined. To visually assess self‐healing of p(TFEMA/*n*BA) copolymers, Figure 1B illustrates optical images of B1) undamaged, B2) damaged, and B3) self‐healed films composed of 50/50 TFEMA/*n*BA monomer molar ratio. When p(TFEMA/*n*BA) copolymer composition is tuned to 45/55, 95 (±4)% of stress, strain, and Young's modulus (*E*) recovery is observed (Figure S1A1–A4, Supporting Information). However, for 50/50 p(TFEMA/*n*BA) ≈75 (±5)% of recovery is observed compared to the undamaged state with the tensile strain ≈700%, maximum stress at break ≈7.3 MPa, and Young's modulus (*E*) of 55.6 MPa (Figure S1B1–B4, Supporting Information). Outside the 45/55–50/50 monomer molar range, no self‐healing is observed (Figure S1C1–C4,D1–D4, Supporting Information). The sensitivity to monomer molar ratio in F‐containing acrylic copolymers formulated hypothesis that in addition to monomer molar ratio, monomer sequencing as well as stereochemistry of neighboring chains may impact inter‐ and/or intramacromolecular interactions, thus affect molecular events and macroscopic viscoelastic response during damage‐repair cycle which may or may not lead to the recovery of mechanical properties.

To test this hypothesis, we examined through‐space interactions of CF_3_ and CH_2_/CH_3_ groups of resonances detected in 2D ^19^F and ^1^H 2D Nuclear Overhauser Effect Spectroscopy (NOESY) as well through bond resonances in 2D^1^H Correlated Spectroscopy (COSY) NMR.^[^ ^4^, ^5^, ^6^, ^7^ ^]^ Figure 1C illustrates 2D ^19^F NOESY spectra of C1) undamaged, C2) damaged, and C3) self‐healed p(TFEMA/*n*BA) copolymers, which exhibit six resonances at (−72.67, −74.48), (−72.84, −73.86), (−73.03, −73.29), (−73.26, −72.72), (−73.46, −72.15), and (−73.66, −71.57) ppm with alternating negative (blue) and positive (red) intensities (Table S1, Supporting Information, provides a summary of all resonances). These resonances are attributed to terminal CF_3_ groups on TFEMA units which in 1D ^19^F NMR spectrum is represented by a singlet at −73.4 ppm (Figure S2, Supporting Information).^[^ ^8^ ^]^ Upon mechanical damage the six resonances due to through‐space interactions inverse their intensities where the positive (red) resonances in undamaged state (Figure 1C1) become negative (blue) in damage state (Figure 1C2), but upon self‐healing (Figure 1C3) return to the initial undamaged state values. At the same time, the −73.4 ppm singlet in 1D experiment increases by ≈15% upon mechanical damage and return to the same intensity upon autonomous repair (Figure S4, Supporting Information). Since 2D ^19^F NOESY experiments detect through‐space interactions, the crosspeak signals (Figure 1C1–C3) arise from the nuclei in close proximity (< 6 Å)^[^ ^9^ ^]^ attributed to crossrelaxations of CF_3_ units or CF_3_ chemical exchange with CH_2_/CH_3_ moieties. The positive diagonal resonance at −73.2 ppm (Figure 1C1–C3) is attributed to conformational/rotational changes of CF_3_ groups due to F nucleus exchange energy with H and F magnetic environments capable of modulating magnetic response of F nucleus. The intensity differences are attributed to the magnitude of magnetization transfer which also depends on the exchange rates and relaxation times. The presence of elliptical ridges results from the reorientation angles of CF_3_ entities which for 2D exchange C─H bonds was observed in 20°–40° range,^[^ ^10^ ^]^ whereas inversion of their intensities during damage‐repair cycle (Figure 1C1–C3) is due to F^…^H heterogenous asymmetric exchange of magnetization during tumbling of magnetically active atoms of the side groups.

If the phase inversions of ^19^F resonances during damage‐repair cycle result from alternations of the CF_3_ surroundings of adjacent side groups of the same or neighboring chain side groups, these changes will also impact ^1^H through space resonances. This is reflected in 2D ^1^H NOESY NMR spectra shown in Figure 1D1–D3, where ^1^H resonance intensities at (a1–a3: 1.88, 1.39)/(b1–b3: 1.39, 1.88) increase by ≈22% upon mechanical damage due to closer proximity of backbone CH_2_ and side‐CH_2_ groups on *n*BA which parallel 2D ^19^F NOESY NMR spectra changes. The absence of (1.88, 1.39)/(1.39, 1.88) resonances in ^1^H 2D COSY NMR (Figure S6, Supporting Information) indicates that CH_2_/CH_3_ interactions are not through bond interactions. Upon self‐healing ^1^H resonances return to their original values (resonances a1/a2/a3 and b1/b2/b3; Figure 1D1–D3). The (a1–a3: 1.88, 1.39)/(b1–b3: 1.39, 1.88) resonance may be attributed to interactions of *α*‐CH_3_ of TFEMA (1.39 ppm) with backbone CH_2_ (1.88 ppm) resulting from the interdigitated chain conformational changes. For p(TFEMA/*n*BA) nonself‐healable copolymer with 40/60 TFEMA/*n*BA molar ratio, 2D^1^H NOESY (Figure S5A1–A3, Supporting Information) and 2D^1^H COSY NMR spectra (Figure S5B1–B3, Supporting Information) exhibit no intensity changes upon mechanical damage. Furthermore, the resonances in 2D ^19^F NOESY NMR spectra show no phase inversion. For 60/40 TFEMA/*n*BA molar ratio copolymer, the same results are observed in 2D^1^H NOESY (Figure S5D1–D3, Supporting Information) and COSY (Figure S5E1–E3, Supporting Information) NMR spectra, but 2D ^19^F NOESY spectra exhibit phase inversion of resonances when going from undamaged (Figure S5F1, Supporting Information) to damaged (Figure S5F2, Supporting Information) state, which remain unchanged upon self‐healing (Figure S5F3, Supporting Information). For self‐healable copolymer compositions the phase inversion is reversible (Figure 1C1–C3).

**Figure 2:**
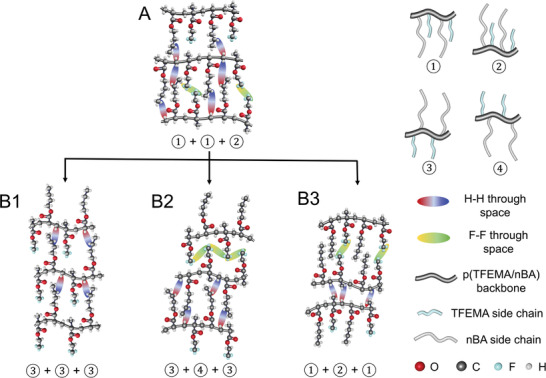
A) Chain conformations and through‐space interactions prior mechanical damage and after self‐repair: B1) preferantially syndiotactic conformations, B2) preferantially syndiotactic conformations, and B3) preferentially isotactic conformations. ①, ②, ③, and ④ represent conformations of individual chains with respect to their neighboring chains, not individual chain conformations.

The analysis of 2D^1^H and ^19^F NOESY NMR data indicates that H···H and F···F through space interactions of copolymer chains in an undamaged state form preferentially isotactic conformations depicted in **Figure** **2**A, where ①, ②, ③, and ④ represent possible tacticities with respect to neighboring chains. Upon mechanical damage the H···H and F···F through space interchain interactions are altered due to the formation of preferentially syndiotactic topologies (Figure 2B1–B3) represented by a combination of (③+③+③), (③+④+③), and (①+②+①) tacticities. Each tacticity contributes to interchain forces due to D‐D, D‐ID, and ID‐ID interactions in damage state, and being energetically unfavorable, results in quasi‐nonequilibrium state. Pronounced CF_3_···F_3_C interactions resulting from highly polar CF_3_ bonds alter stereoconfiguration and chain conformations during damage‐repair cycle and external D‐D or D‐ID perturbations cause the intensity of the CF_3_ resonances to decrease due to macromolecular chain “compression,” which is reflected in the intensity inversions in 2D ^19^F NOESY NMR resonances due to conformational changes (Figure 1C1–C3). Similarly, since ^19^F is considerably more sensitive to local magnetic environment than ^1^H, ≈15% decrease of ^19^F singlet in 1D NMR (Figure S4, Supporting Information) upon damage is attributed to the shortening of T_2_ relaxation times.^[^ ^11^ ^]^ As macromolecular segments are in closer proximity upon returning to self‐healed interdigitated equilibrium, “decompression” will lead to longer interchain distances.

**Figure 3:**
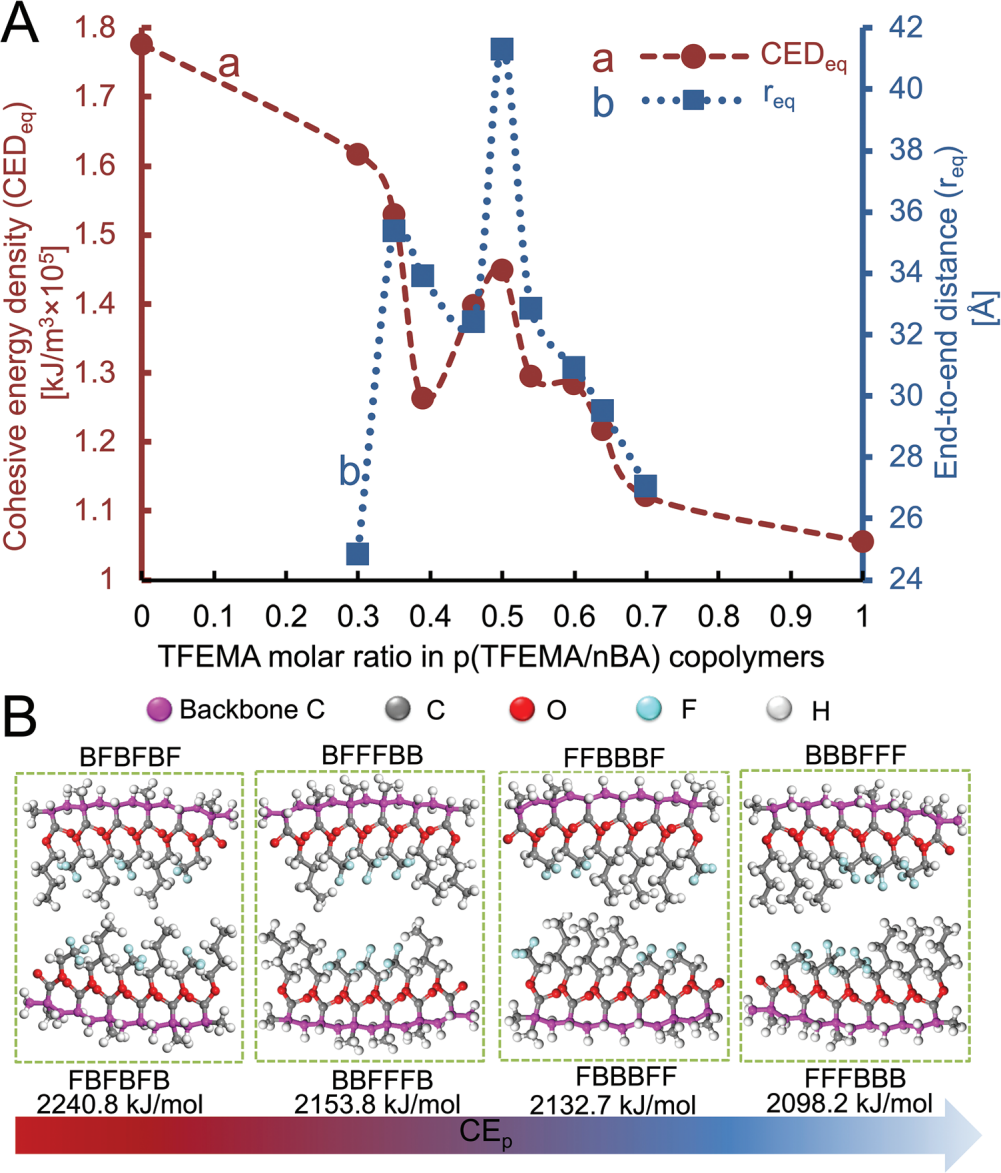
A) Cohesive energy densities at equilibrium (CED_eq_) (curve a) and average end‐to‐end equilibrium distances (*r*_eq_) (curve b) plotted as a function of molar fraction of TFEMA in p(TFEMA/*n*BA) copolymers. (B) Cohesive energies (CE_p_) for selected hexads combinations: BFBFBF/FBFBFB, BFFFBB/BBFFFB, FFBBBF/FBBBFF, and BBBFFF/FFFBBB.

If mobility of side groups of p(TFEMA/*n*BA) copolymer reflected in 2D ^19^F and ^1^H NOESY NMR intensity changes is responsible for regaining mechanical properties in the relatively narrow monomer compositional range, the question is what specific inter‐ or/and intrachain interactions main contributors to self‐healing events are and how they impact copolymer backbone mobility. Using molecular dynamic (MD) simulations, we examined cohesive energy density (CED) changes as a function of copolymer compositions in the 30/70 to 70/30 TFEMA/*n*BA monomer molar ratio range. **Figure** **3** plots CEDs values and end‐to end distances (*r*) as a function of the copolymer composition. As shown, the CED values at equilibrium (CED_eq_) (curve a) and end‐to‐end distances (*r*_eq_) (curve b) reach maxima at 1.45 kJ m^−3^ × 10^5^ and ≈41 Å, respectively, near 50/50 TFEMA/*n*BA molar ratio which corresponds to experimentally determined 45/55 to 55/45 self‐healable monomer compositional range. For nonself‐healable 40/60 and 60/40 TFEMA/*n*BA molar ratios, the CED_eq_ and r_eq_ values decrease to ≈1.26 kJ m^−3^ × 10^5^ and ≈33.9 Å (40/60 TFEMA/*n*BA) and 1.28 kJ m^−3^ × 10^5^ and ≈30.9 Å, respectively. These data show that predominantly alternating/random copolymer compositions with extended‐helical topologies enhance directionality of vdW forces, thus enhancing interchain interactions reflected in the higher CED_eq_ and *r*_eq_ values.

During statistical copolymerization it is anticipated that even for monomers with similar reactivity ratios, certain fraction of copolymerized repeating monomer units’ triads or even larger size blocks will form. To elucidate the influence of monomer sequencing on self‐healing, model MD simulations were conducted in which cohesive energies (CE_p_) of combinations of selective F and B hexad sequences (where: F = TFEMA and B = *n*BA) were determined. As shown in Figure 3B, alternating BFBFBF/BFBFBF pair (1:1) exhibits the highest CE_p_ values (2240.5 kJ mol^−1^), whereas CE_p_s for more “blocky” pair combinations are lower, further supporting the experimental evidence that D‐D and D‐ID interactions are the strongest for preferentially alternating copolymer sequences, thus facilitating autonomous self‐healing (Figure 3B).

**Figure 4:**
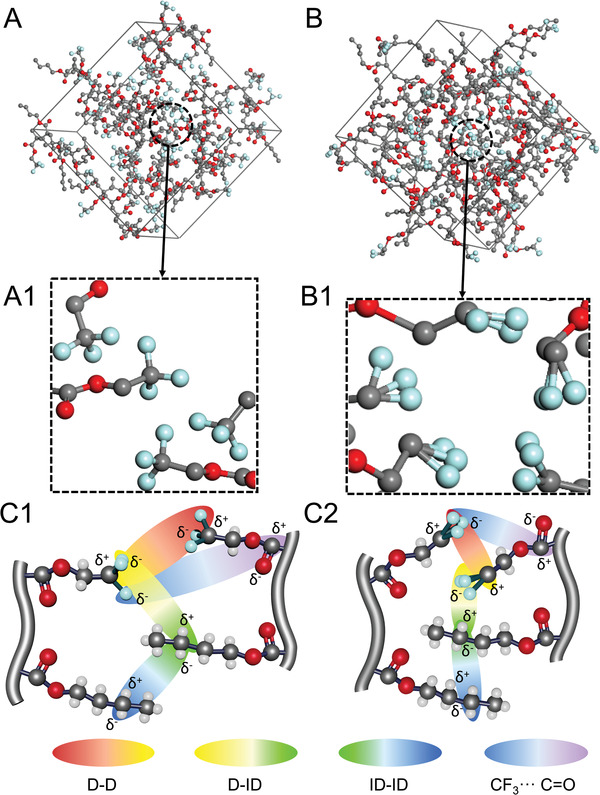
Unit cells used in MD simulations for hexads at A) 1.125 g cm^−3^ and B) 3.36 g cm^−3^ densities. (A1) and (B1) are the enlarged extracts from (A) and (B). (C1) and (C2) depict dipole‐dipole (D‐D), dipole‐induced dipole (D‐ID), and induced‐dipole‐induced‐dipole (ID‐ID) forces resulting from of CF_3_⋅⋅⋅CF_3_, CH_2_⋅⋅⋅CH_2_, and CF_3_⋅⋅⋅C=O interactions in C1) equilibrium and C2) quasi‐nonequilibrium compressed states.

If alternating copolymer topology favors energetically more stable conformations resulting from the high polarity of C─F bonds resulting in dipolar interactions, in addition to CF_3_⋅⋅⋅F_3_C interactions of neighboring chains, CF_3_⋅⋅⋅C═O interactions are anticipated. For example, for porcine pancreatic elastase and an inhibitor of the trifluoromethyl peptide interactions, the CF_3_⋅⋅⋅C═O interactions with distances smaller than the sum of vdW radii of C and F ≈3.3 Å were determined,^[^ ^12^, ^13^ ^]^ which correspond to a hemispherical volume of ≈35.9 Å^3^. If during copolymer damage “compression” of neighboring chains occurs, it is expected that the CF_3_⋅⋅⋅C═O and CF_3_⋅⋅⋅F_3_C distances will be diminished and the hemispherical volumes will also decrease. The results of model MD simulations for hexads with 1.125 g cm^−3^ (**Figure** **4**A) and compressed to 3.36 g cm^−3^ (Figure 4B) densities indicate that for alternating BFBFBF hexads, the average CF_3_⋅⋅⋅C═O distance is ≈6.1 Å, which corresponds to ≈227.0 Å^3^, but in the quasi‐nonequilibrium “compressed” state this distance is reduced to ≈3.86 Å. Also, the average CF_3_⋅⋅⋅F_3_C distance of ≈4.45 Å (88.1 Å^3^) is reduced for compressed hexads to ≈2.85 Å (≈23.1 Å^3^), whereas for CF_3_⋅⋅⋅C═O average distance is reduced from ≈6.1 to ≈3.86 Å. These changes are illustrated in Figure 4 which shows the results of MD simulations for hexads under uncompressed (A; 1.125 g cm^−3^) and compressed conditions (B; 3.36 g cm^−3^). Upon compression, MD simulations show that there are also significant changes in the vicinity of CF_3_ and CH_2_/CH_3_ groups which were experimentally observed in 2D ^19^F and ^1^H NOESY NMR experiments for self‐healable compositions (Figure 1C1–C3,D1–D3). When copolymer is damaged (compression state) CF_3_ units undergo structural rearrangements from tetrahedral (Figure 4A1) with an average F─C─F bond angle of 109.1° to almost off‐planar geometry (Figure 4B1) and an average F─C─F bond angle of 32.8°. The latter is reflected in elliptical ridges in 2D^1^H and ^19^F NOESY NMR spectra. These local symmetry changes within the vicinity of CF_3_ moieties are attributed to D‐D (Keesom), D‐ID (Debye), and ID‐ID (London dispersion) changes. While D‐D interactions result from permanent dipoles of CF_3_ bonds (Figure 4C1,C2) for equilibrated and compressed states, respectively, the D‐ID and ID‐ID forces arise from fluctuations in the distribution of electrons in the surrounding CH_2_/CH_3_ units also reflected in the NMR intensity changes (Figure 1D1–D3).

It is also useful to consider how D‐D, D‐ID, and ID‐ID interactions impact overall entropic energy (Δ*S*_s_) stored during mechanical damage of copolymers. We conducted dynamic mechanical analysis experiments for copolymer films with 40/60 to 60/40 monomer molar ratios in which, by measuring the junction densities (*v*_j_) and viscoelastic length transitions transitions,^[^ ^14^, ^15^ ^]^ Δ*S*_s_ values were determined as a function of copolymer composition (Table S2, Supporting Information). For self‐healable copolymers in the ≈50/50 TFEMA/*n*BA molar ratio range the highest Δ*S*_s_ values are observed, further supporting the evidence that the monomer molar ratio and copolymer topology facilitate strong dipolar interactions that facilitate self‐healing.

## 3 Conclusions

In summary, these studies show a route for copolymerizing fluorinated copolymers in which interchain interactions resulting from dipolar forces facilitate autonomous self‐healing upon mechanical damage. Due to higher rotational energy of CF_3_ groups (≈20 kJ mol^−1^) compared to CH_2_CH_3_ (5–10 kJ mol^−1^)^[^ ^16^ ^]^ in p(TFEMA/*n*BA), CF_3_ groups are strong contributors to stereoconfiguration and chain conformational energy changes during damage‐repair cycle. These findings also suggest other polymer properties including thermal conductivity may be favorably altered by the presence of extended chains and restricting angular freedom along polymer chains due to higher dipolar forces. Not only chain extension but also torsions of repeating units, sequence of repeating units, and local repeating unit inter‐ and intrachain interactions should be well thought out when designing commodity copolymers with unique properties. Although a number of outstanding self‐healing polymers have been developed,^[^ ^17^ ^]^ the use of dipolar interactions instead of physicochemical modifications may be advantages for commodity copolymers.

## Conflict of Interest

The authors declare no conflict of interest.

## Author Contribution

The experiment was designed by M.W.U. and S.W. and conducted by S.W. Data analysis was performed by S.W. and M.W.U. M.W.U and S.W. wrote the paper.

## Supporting information

Supporting Information

Supplemental Movie 1

## Data Availability

All data needed to evaluate the conclusions in the paper are present in the paper or the Supporting Information. Additional information may be available from the corresponding author upon reasonable request.
