# Supplementary material for: Self‐Healable Fluorinated Copolymers Governed by Dipolar Interactions
Source: Adv Sci (Weinh). 2021 Jul 6;8(17):2101399. doi: 10.1002/advs.202101399 (PMC8425892; doi:10.1002/advs.202101399)
Supplement: Supplementary file 1 — Supporting Information [file ADVS-8-2101399-s002.pdf]

© 2021 Wiley-VCH GmbH

ADVANCED SCIENCE
Open Access

Supporting Information

for *Adv. Sci.*, DOI: 10.1002/advs.202101399

Self-Healable Fluorinated Copolymers Governed by Dipolar Interactions

*Siyang Wang and Marek W. Urban**

Supplementary Information

Self-Healable Fluorinated Copolymers Governed by Dipolar Interactions

Siyang Wang and Marek W. Urban*

Department of Materials Science and Engineering

Clemson University, Clemson, SC 29634

* corresponding author (mareku@clemson.edu)

Table of Contents

Materials and Methods
Supplementary Discussion
Supplementary Figures: S1 to S8
Supplementary Tables: S1 to S4
Captions for Video S1
Supplementary Video

Materials and Methods

Materials

2,2,2-trifluoroethyl methacrylate (TFEMA) (stabilized with monomethyl ether hydroquinone (MEHQ)) and n-butyl acrylate (nBA) (stabilized with MEHQ), methanol (MeOH) and tetrahydrofuran (THF) were purchased from Thermo Fisher-Scientific. 2,2'-Azobis(2-methylpropionitrile) (AIBN) was purchased from Sigma-Aldrich. All monomers were purified before polymerization by passing through neutral activated aluminum oxide (Sigma-Aldrich) column.

Methods

Statistical p(TFEMA/nBA) copolymers were synthesized using free radical polymerization. In a typical experiment, each TFEMA/nBA monomer molar ratios were dissolved in THF at the concentration of 1.5g/mL, and 0.05 wt% of AIBN was added to the round bottom flask. N_2 gas was purged for 30min prior placing the flask into a 75 ºC oil bath. Polymerization was conducted at 75ºC for 8 hrs while stirring at 400rpm. The resulting reaction p(TFEMA/nBA) copolymer was diluted with 10mL THF and precipitated out in MeOH. The last step involved centrifuging at 9000 rpm for 5min and removal of upper layer solvent, followed by drying overnight at 75 ºC in vacuum oven.

p(TFEMA/nBA) copolymer films were prepared by dissolving copolymers in THF at the concentration of 0.8g/mL, and dried in Teflon^{TM} mold placed in sealed container under ambient conditions for 2 days, followed by drying at 75 ºC in vacuum oven for 24 hrs.

Analytical methods

Gel permeation chromatography (GPC) measurements were conducted on Tosoh EcoSEC GPC calibrated with HPLC grade poly(methyl methacrylate) (PMMA) standards using refractive index (RI) detector. Prior each measurement, copolymers were dissolved overnight in HPLC grade THF (purchased from Fisher Scientific) at the concentration of 1.5mg/ml followed by passing through 0.2µm PTFE filter.

Differential scanning calorimetry (DSC) measurements were performed on Q100 series TA Instruments. Typical heating/cooling cycle of DSC measurements were conducted at 20 ºC/min rate from -100 ºC to 150 ºC. Data analysis was conducted by TA Universal Analysis software.

Dynamic light scattering (DLS) experiments were conducted on Zetasizer Nano-Zen 3600 manufactured by Malvern Instruments. The same sample preparation were utilized as in 2D NMR experiments.

Optical images of self-healing of p(TFEMA/nBA) copolymer films were recorded using RENISHAW inVia^{TM} Raman microscope (20×).

Mechanical properties

Self-healing efficiencies and mechanical properties of copolymers before damage and after self-healing were measured using Instron Model 5500R1125 (stress-strain). In a typical experiment, the gauge length and the strain rate were set to 1.5cm and 80mm/min respectively. To determine the self-healing capability of p(TFEMA/nBA) copolymers, 0.3×1.5×0.2 cm (W×L×T) films were damaged using stainless-steel razorblades forming cuts 50µm in width and ~60 µm in depth. These damaged films were allowed to heal under ambient conditions at room temperature (25°C) for 48hrs. The same stress-strain measurements were performed for undamaged and damaged samples. Stress-strain measurements were repeated 10 times for each copolymer composition.

^{1}H and ^{19}F NMR spectroscopic measurements were conducted on 300MHz JEOL Model ECX-300 spectrometer with 1.5s relaxation delay and each spectrum represents 64 added scans. 2D ^{19}F NOESY experiments were performed on the same JEOL spectrometer with 1.5s relaxation delay, 0.5s mixing time and each spectrum represents 8 coadded scans. 2D ^{1}H NOESY NMR experiments were conducted on 500MHz AVANCE NEO spectrometer with 1.5s relaxation delay, 0.5s mixing time and 8 coadded

scans collected for all undamaged, damaged, and self-healed samples. 2D ^1H COSY spectra represents 32 coadded scans.

NMR samples were prepared at the concentration of 1mg/ml in $CDCl_3$ for 1D ^1H and ^{19}F NMR, and 15mg/ml for 2D ^1H COSY, NOESY, and ^{19}F NOESY NMR. 2D ^1H COSY, ^1H NOESY, and ^{19}F NOESY NMR spectra were processed using MestReNova software. Undamaged, damaged, and healed 2D ^1H NOESY NMR spectra were normalized to (4.37, 0.97) assigning to -OC**H_2** and α-C**H_3** of pTFEMA. ^{19}F NMR spectra were processed using TopSpin software. 2D NMR experiments, p(TFEMA/nBA) film (5×5×0.5 mm) were damaged with 20×20 cuts on both top and bottom sides prior dissolving in $CDCl_3$ at the concentration of 15mg/ml for 10min without agitation, resulting in ~ 42% ratio of damaged area.[1] Healed copolymers were prepared using identical damaged sample which allowed to heal at 37 ºC for 24hrs before dissolving for 10min in $CDCl_3$ at the concentration of 15mg/ml without agitation.

Dynamic mechanical analysis (DMA) was conducted using a TA Instruments Q800 DMA in strain control mode. Rectangular films with the width ~5.5mm and thickness ~0.5mm, and 10mm gauge length at room temperature were tested using 10 µm amplitude, 10 Hz frequency, force track 125%, at a heating rate of 2 ºC/min as standard experimental conditions. Table S2 summarizes junction density (v_j), and stored entropy and (ΔS_s) for 40F/60B, 45F/55B, 50F/50B, 55/45B, and 60F/40B p(TFEMA/nBA).

Molecular dynamic (MD) simulations were conducted using Materials Studio software (v 5.5.0.0) (distributed by BIOVIA). Amorphous cell module and DRIEDING force field under isothermal (NVT) conditions were utilized to determine cohesive energy density (CED) as a function of copolymer compositions. For each p(TFEMA/nBA) composition, seven p(TFEMA/nBA) copolymer chains containing 60 monomer units each were placed into an amorphous unit cell at the density of 1.125 g/cm^3. The pTFEMA and pnBA homopolymer densities were 1.181 g/cm^3 and 1.087 g/cm^3. According to the literature,[2] during free radical propagation, stereochemistries of -$COOCH_3$ in MMA and -$COOCH_2CF_3$ in TFEMA side chains are indistinguishable. We conducted parallel

studies with the same reactivity ratio as MMA and nBA system, which are 2.60 for r_{TFEMA} and 0.39 for r_{nBA}. Prior to equilibration, copolymers in each unit cell were geometrically optimized using 1000. Subsequently, each cell was allowed to equilibrate for 100 psec (NVT, isothermal at 298K, at a time-step of 0.33 fsec, Berendsen thermostat) to obtain primary values of minimized energies. The cohesive energy densities for equilibrated compositions (CED_{eq}), vdW forces densities at equilibrium (vdW_{eq}) were calculated using Forcite cohesive energy density module.

Supplementary Discussion

In an effort to eliminate the possibility that sample preparation for 2D ^{19}F and ^{1}H NMR experiments may have impacted p(TFEMA/nBA) copolymer conditions, a series of control experiments were conducted on filtered undamaged and damaged copolymer specimens (filter pore size: 0.2μm). As shown in Figure S3, A and B, 2D ^{19}F NOESY NMR spectra show identical sequence of resonances for both undamaged and damaged samples. DLS measurements showed the same particle size distribution for both filtered samples. This control experiment shows that the inversible resonances result from interactions within the insolubilized parts of copolymers.

A typical 2D NOESY or COSY NMR experiment takes 100 min. To eliminate the possibility that inter-chain interactions may be altered during solvation process when dispersing p(TFEMA/nBA) copolymers in $CDCl_3$, a series of controlled experiments were conducted using DLS capable of measuring 10-1000 nm particle size. The DLS sample preparation paralleled 2D NOESY or COSY NMR experimental conditions and showed that after 100 min without agitation the particle size exceeds the instrument upper detection limits.

For both non-self-healable copolymers with TFEMA/nBA molar ratios of 40/60 and 60/40, the intensities of cross peak (1.88, 1.39)/(1.39, 1.88) in 2D ^{1}H NOESY NMR spectra (Figure S5 A-A'', C-C'' respectively) remain constant within experimental error. The phase sequences resume identical for 2D ^{19}F NOESY NMR spectra (Figure S5 C-C'') across the undamaged, damaged and post-damaged process for 40/60 copolymers, whereas for 60/40 copolymers, the phase sequences (Figure S5 F-F'') are reversed when comparing undamaged and damaged samples, the phases of post-damaged

samples after two days are the same as the damaged one due to the lack of self-healing capability.

The following normalization resonance (4.37, 0.97) in 2D ^{1}H NOESY NMR were used, instead of previously mentioned assigning 0.97ppm assigned to α-C**H₃** in pTFEMA, terminal -C**H₃** on the side chain of nBA. In this case, the requirement of unchanged interactions through damage-repair cycles may not be satisfied. However, by comparing the pre-normalized spectra of undamaged, damaged, and healed specimens, the integration of (4.37, 0.97) resonance are consistent, which eliminated this possibility. Table S2 summarizes monomer feed (f) and actual (F) ratios in p(TFEMA/nBA) copolymers which were determined using ^{1}H NMR spectroscopy.[3]

Junction densities (v_j), stored entropy (ΔS_s), and mol. wt. between junction points (M_j) were determined using dynamic mechanical analysis (DMA). In a typical DMA experiment, storage modulus (E'), loss modulus (E''), tan δ (log(E'')/log(E')), and viscoelastic length transitions (VLT) as a function of temperature were obtained. Using experimental VLT values from a single DMA measurement and applying rubber elasticity theory allows us to calculate the v_j using the following relationship $v_j = \sigma_R$ / [RT (α^{-1}/α^2)]; where: σ_R is the retractive stress and α is the elongation ratio (L/L_0) obtained from the DMA analysis. Using $\Delta S = -(Rv_j /2) \times [\alpha^2+2/\alpha-3]$[4], this approach also allows determination of stored entropy $\Delta S_s = -T_{\varepsilon max}S_{\varepsilon max} + T_iS_i$; where: $T_{\varepsilon max}$ and $S_{\varepsilon max}$ are temperature and entropy at max elongation (ε_{max}), and T_i and S_i represent values before elongation.[5] These ΔSs and v_j are shown as a function of copolymer composition (Table S3).

To determine the impact of monomer distribution on CED and self-healing properties, NPT and NVT simulations were performed on a series of TFEMA/nBA hextads. In a typical experiment, 14 identical hextads were placed into a unit cell at 0 GPa with the density of 1.125 g/cm^3. Afterwards, all the hextads were allowed to reach optimum packing density under NPT equilibration for 100ps. Subsequently, the hextads were loaded into a unit cell with the equilibrium densities calculated via NPT ensemble, followed by 100ps isothermal equilibration via NVT. The average cohesive energy (CE_p) values were calculated.

Figure S7 illustrates multiple damage-repair cycles conducted on p(TFEMA/nBA) copolymer films with TFEMA/nBA = 45/55 monomer molar ratio films under ambient conditions. Each damage-repair cycle represents approximately 1.5~2 hrs between subsequent cuts.

Figure S8 illustrates the GPC traces and Table S4 summarizes molecular weights and dispersity of selected p(TFEMA/nBA) copolymers.

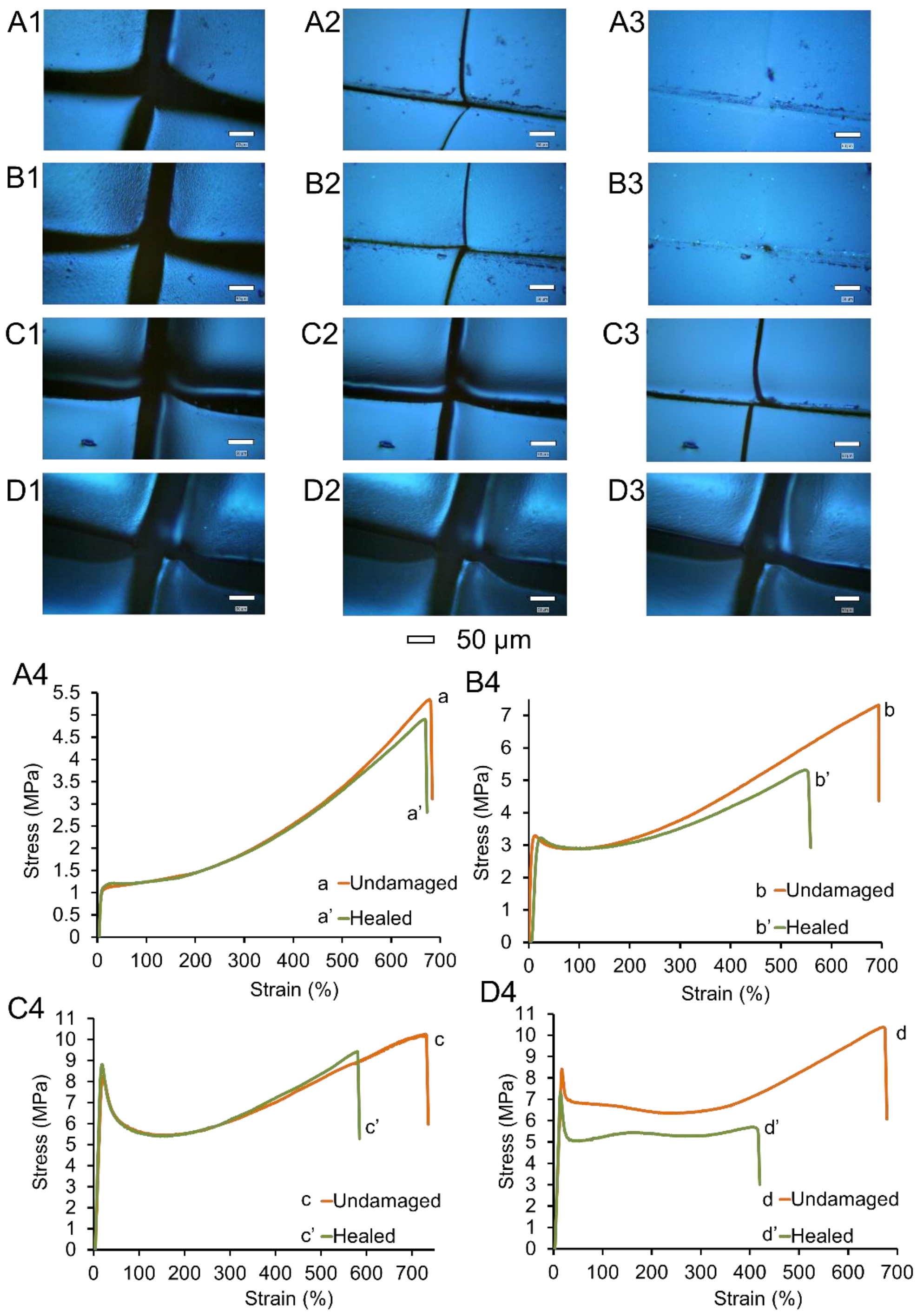

Figure S1 Optical images of undamaged (A1-D1), 1hr after damage (A2-D2), self-healed (2 days, A3-B3) and after damaged (2-5 days, C3-D3) p(TFEMA/nBA) copolymer films composed of 45/55 (A series), 50/50 (B series), 56/44 (C series) and 60/40 (D series) TFEMA/nBA molar ratios; stress-strain curves (A4-D4) for undamaged (curves a-d) and after self-healed (curves a'-d').

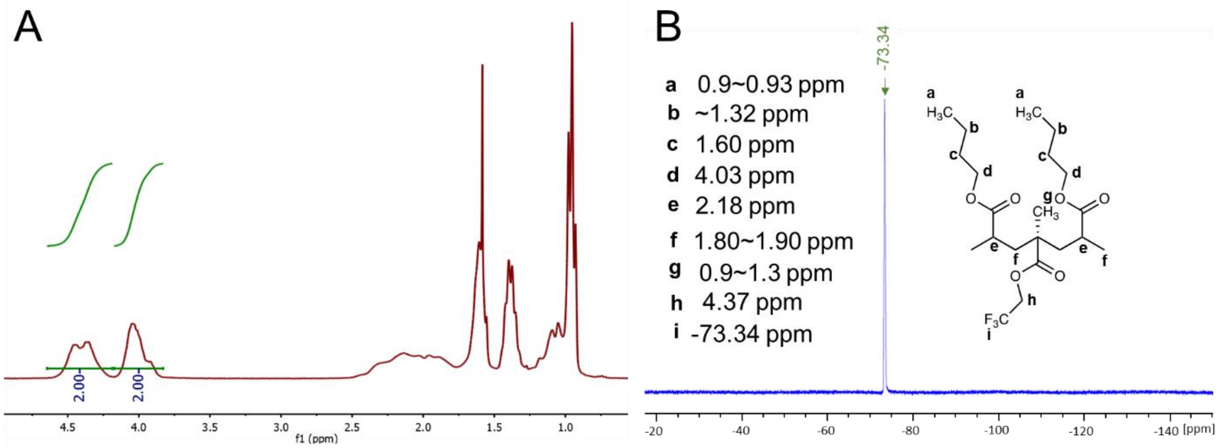

Figure S2 (A) ^{1}H and (B) ^{19}F NMR spectra of p(TFEMA/NBA) copolymer with 50/50 monomer molar ratios copolymer and corresponding resonance assignments.

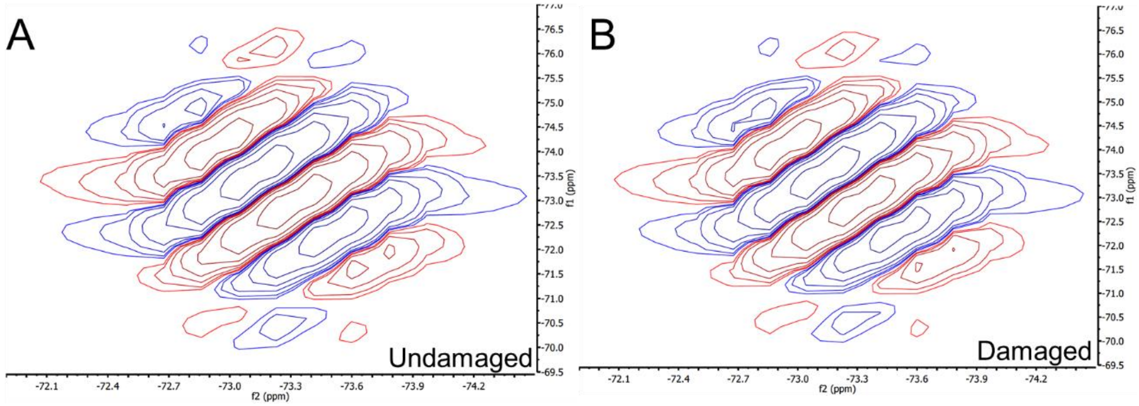

Figure S3 2D ^{19}F NOESY NMR spectra of copolymer films with 50/50 TFEMA/nBA monomer molar ratios for filtered (A) undamaged and (B) damaged film specimens.

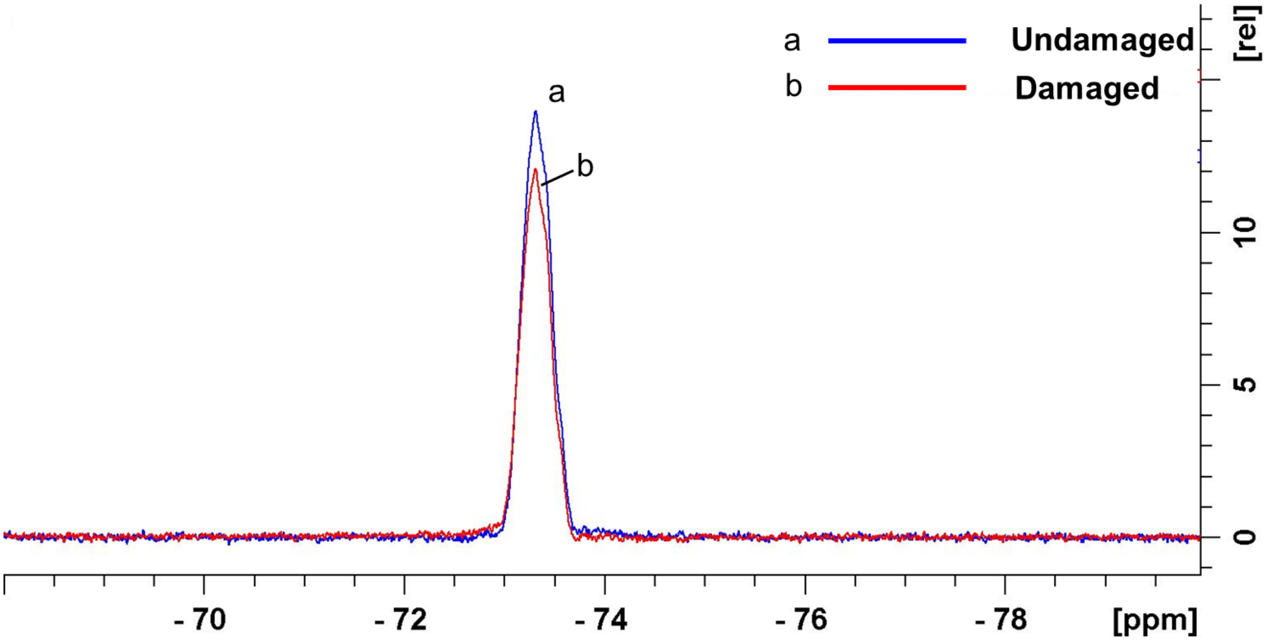

Figure S4 1D ^{19}F NMR spectra of undamaged (a) and damaged (b) p(TFEMA/nBA) copolymer with 50/50 monomer molar ratio.

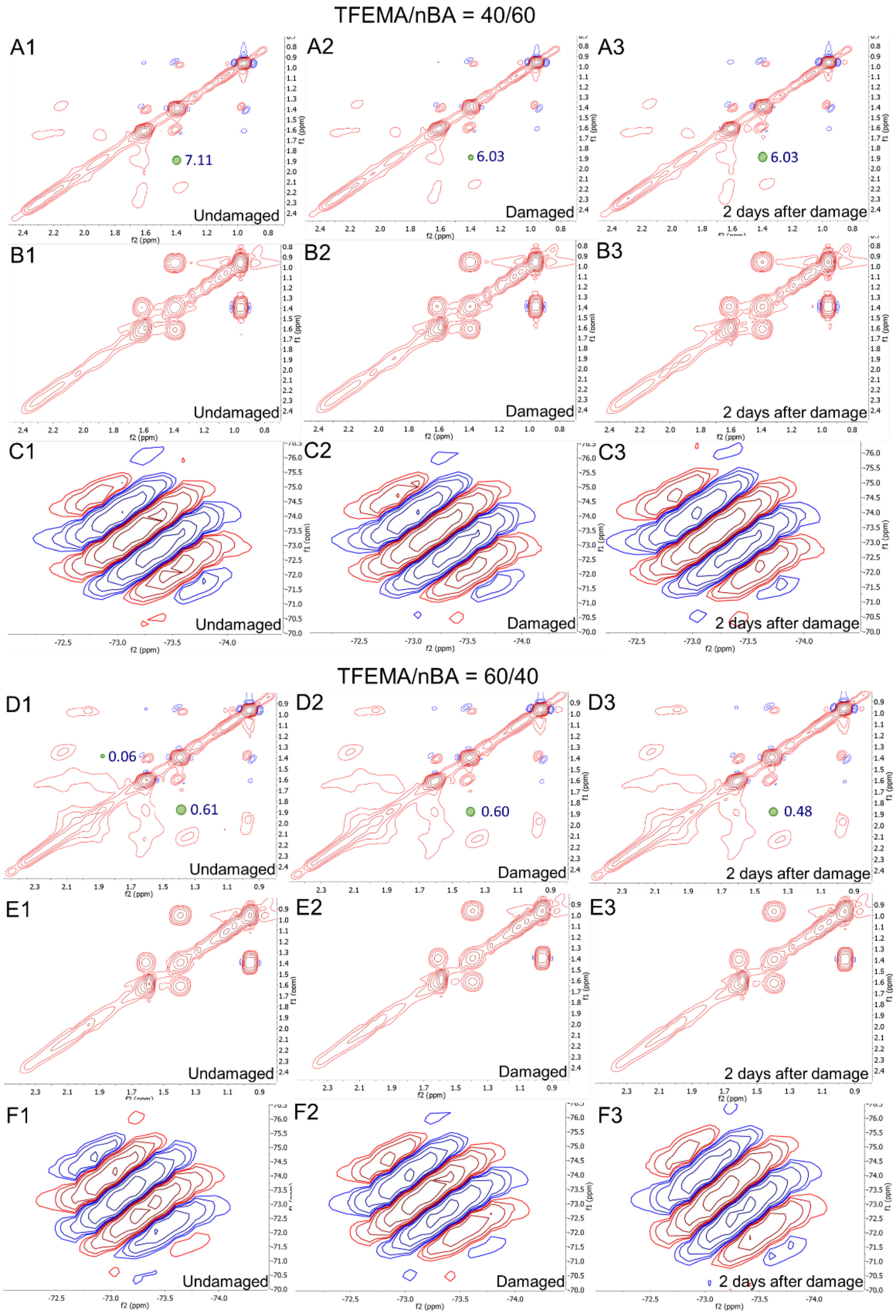

Figure S5 2D ^{1}H NOESY NMR spectra of p(TFEMA/nBA) copolymer with a 40/60 TFEMA/nBA monomer molar ratio: undamaged (A1), damaged (A2) and 2 days after damaged (A3); 2D ^{1}H COSY NMR spectra of p(TFEMA/nBA) copolymer with a 40/60 TFEMA/nBA monomer molar ratio: undamaged (B1), damaged (B2) and 2 days after

damaged (B3); 2D ^{19}F NOESY NMR spectra of of p(TFEMA/nBA) copolymer with a 40/60 TFEMA/nBA monomer molar ratio: undamaged (C1), damaged (C2) and 2 days after damaged (C3). 2D ^{1}H NOESY spectra of p(TFEMA/nBA) copolymer with a 60/40 TFEMA/nBA monomer molar ratio: 2D ^{1}H NOESY NMR spectra of p(TFEMA/nBA) copolymer with a 60/40 TFEMA/nBA monomer molar ratio: undamaged (D1), damaged, (D2) and 2 days after damaged (D3); 2D ^{1}H COSY NMR spectra of p(TFEMA/nBA) copolymer with a 60/40 TFEMA/nBA monomer molar ratio: undamaged (E1), damaged (E2), and 2 days after damaged (E3); 2D ^{19}F NOESY NMR spectra of p(TFEMA/nBA) copolymer with a 60/40 TFEMA/nBA monomer molar ratio undamaged (F1), damaged (F2), and 2 days after damaged (F3).

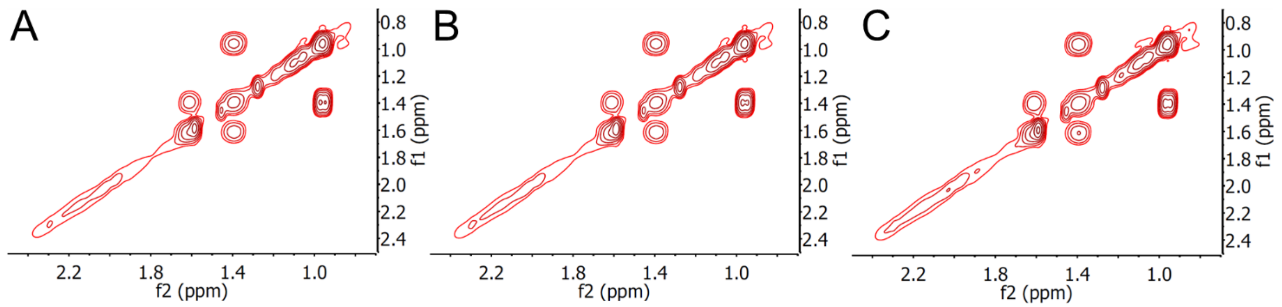

Figure S6 2D ^{1}H COSY NMR spectra of undamaged (A), damaged (B) and self-healed (C) of p(TFEMA/nBA) copolymer with 50/50 TFEMA/nBA monomer molar ratio.

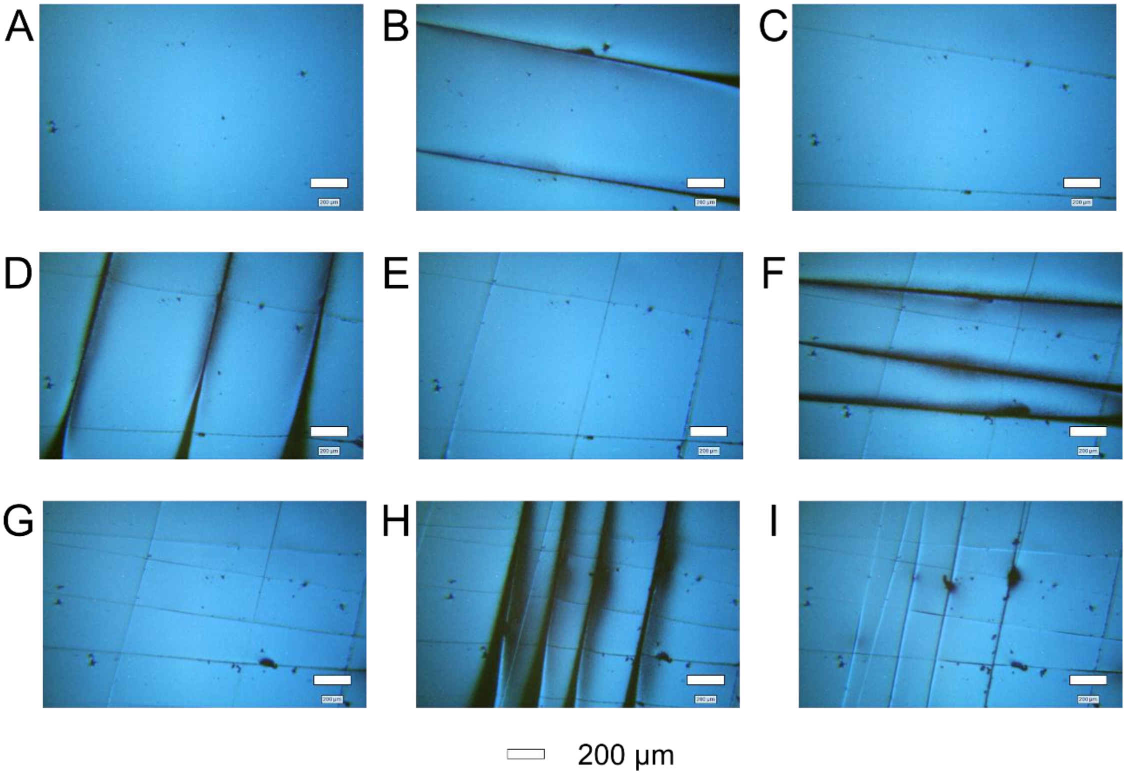

Figure S7 Optical images of p(TFEMA/nBA) copolymer films with a 45/55 monomer molar ratio exposed to multiple cuts in 1.5~2 hrs intervals. (A) undamaged; (B) first damage cycle; (C) self-healed after first cycle of multiple cuts (room temperature); (D) second damage cycle; (E) self-healed after second cycle of multiple cuts; (F) third damage cycle; (G) self-healed after third cycle of multiple cuts; (H) fourth damage cycle; (I) self-healed after fourth cycle of multiple cuts. This process can be repeated multiple times.

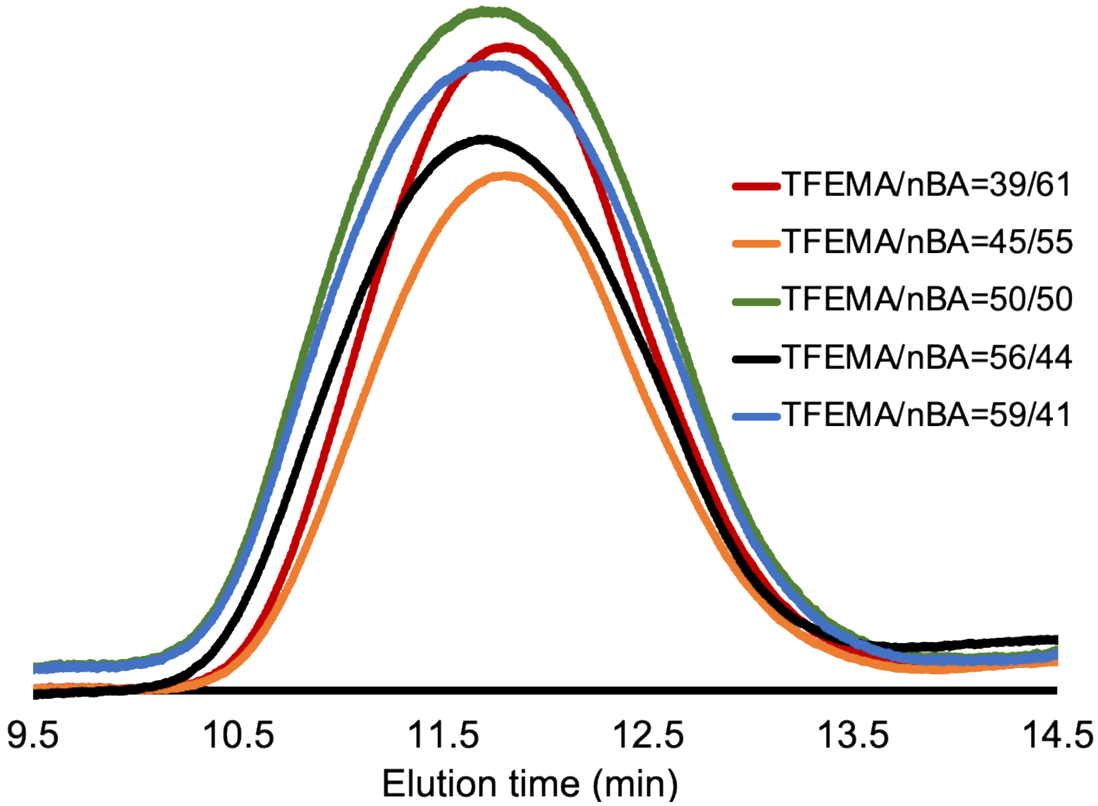

Figure S8 GPC chromatograms for p(TFEMA/nBA) copolymers with different TFEMA/nBA compositions ranging from 39/61 to 59/41.

Table S1 Chemical shifts and intensities of 2D ^{19}F NOESY NMR resonances p(TFEM/nBA) films with 40/60, 50/50, and 60/40 monomer molar ratios. Intensity values correspond to positive and negative intensities in 2D ^{19}F NOESY NMR spectra (Figure 1, B1-B3 (red '+'and blue '-').

		Resonance 1		Resonance 2		Resonance 3		Resonance 4		Resonance 5		Resonance 6	
40F/60B Undamaged	Phase	+		-		+		-		+		-	
		Peak 1	Peak 2	Peak 1	Peak 2	Peak 1	Peak 2	Peak 1	Peak 2	Peak 1	Peak 2	Peak 1	Peak 2
	Chemical shifts	(-72.68, -74.37)	(-72.81, -74.73)	(-72.84, -73.72)	(-73.01, -74.16)	(-73.05, -73.12)	(-73.21, -73.57)	(-73.25, -72.52)	(-73.41, -72.98)	(-73.45, -71.93)	(-73.61, -72.38)	(-73.65, -71.37)	(-73.82, -71.77)
	# of contours	3		5		6		6		5		2	
40F/60B Damaged	Phase	+		-		+		-		+		-	
		Peak 1	Peak 2	Peak 1	Peak 2	Peak 1	Peak 2	Peak 1	Peak 2	Peak 1	Peak 2	Peak 1	Peak 2
	Chemical shifts	(-72.64, -74.33)	(-72.82, -74.70)	(-72.85, -73.68)	(-73.02, -74.12)	(-73.06, -73.08)	(-73.22, -73.54)	(-73.26, -72.49)	(-73.42, -72.94)	(-73.46, -71.90)	(-73.62, -72.34)	(-73.66, -71.33)	(-73.83, -71.73)
	# of contours	3		5		6		6		5		2	
40F/60B 24hrs after damage	Phase	+		-		+		-		+		-	
		Peak 1	Peak 2	Peak 1	Peak 2	Peak 1	Peak 2	Peak 1	Peak 2	Peak 1	Peak 2	Peak 1	Peak 2
	Chemical shifts	(-72.65, -74.18)	(-72.83, -74.55)	(-72.86, -73.53)	(-73.03, -73.97)	(-73.07, -72.93)	(-73.22, -73.38)	(-73.27, -72.33)	(-73.42, -72.79)	(-73.47, -71.75)	(-73.63, -72.19)	(-73.67, -71.21)	(-73.83, -71.60)
	# of contours	3		5		6		6		5		2	
50F/50B Undamaged	Phase	-		+		-		+		-		+	
		Peak 1	Peak 2	Peak 1	Peak 2	Peak 1	Peak 2	Peak 1	Peak 2	Peak 1	Peak 2	Peak 1	Peak 2
	Chemical shifts	(-72.67, -74.48)	(-72.84, -74.87)	(-72.84, -73.86)	(-73.00, -74.32)	(-73.03, -73.29)	(-73.23,-73.77)	(-73.26, -72.72)	(-73.42, -73.19)	(-73.46, -72.15)	(-73.62, -72.58)	(-73.66, -71.57)	(-73.80, -71.93)
	# of contours	3		5		6		6		5		3	
50F/50B Damaged	Phase	+		-		+		-		+		-	
		Peak 1	Peak 2	Peak 1	Peak 2	Peak 1	Peak 2	Peak 1	Peak 2	Peak 1	Peak 2	Peak 1	Peak 2
	Chemical shifts	(-72.64, -74.46)	(-72.81, -74.85)	(-72.84, -73.84)	(-73.01, -74.30)	(-73.04, -73.26)	(-73.20, -73.74)	(-73.23, -72.70)	(-73.43, -73.16)	(-73.43, -72.12)	(-73.59,-72.55)	(-73.67, -71.55)	(-73.81, -71.91)
	# of contours	3		5		6		6		5		3	
50F/50B Healed	Phase	-		+		-		+		-		+	
		Peak 1	Peak 2	Peak 1	Peak 2	Peak 1	Peak 2	Peak 1	Peak 2	Peak 1	Peak 2	Peak 1	Peak 2
	Chemical shifts	(-72.66, -74.37)	(-72.83, -74.77)	(-72.86, -73.77)	(-73.02, -74.25)	(-73.05, -73.22)	(-73.21, -73.70)	(-73.25, -72.66)	(-73.41, -73.12)	(-73.45, -72.07)	(-73.61, -72.48)	(-73.67, -71.44)	(-73.80, -71.79)
	# of contours	3		5		6		6		5		2	
60F/40B Undamaged	Phase	-		+		-		+		-		+	
		Peak 1	Peak 2	Peak 1	Peak 2	Peak 1	Peak 2	Peak 1	Peak 2	Peak 1	Peak 2	Peak 1	Peak 2
	Chemical shifts	(-72.66, -74.22)	(-72.83, -74.65)	(-72.86, -73.64)	(-73.02, -74.14)	(-73.04, -73.10)	(-73.23, -73.62)	(-73.27, -72.58)	(-73.42, -73.07)	(-73.46, -72.02)	(-73.63, -72.45)	(-73.67, -71.42)	(-73.82, -71.72)
	# of contours	3		5		6		6		4		1	
60F/40B Damaged	Phase	+		-		+		-		+		-	
		Peak 1	Peak 2	Peak 1	Peak 2	Peak 1	Peak 2	Peak 1	Peak 2	Peak 1	Peak 2	Peak 1	Peak 2
	Chemical shifts	(-72.66, -74.11)	(-72.83, -74.52)	(-72.86, -73.50)	(-73.02, -73.98)	(-73.05, -72.94)	(-73.21, -73.44)	(-73.25, -72.40)	(-73.44, -72.87)	(-73.47, -71.85)	(-73.60, -72.27)	(-73.68, -71.28)	(-73.82, -72.61)
	# of contours	3		5		6		6		4		1	
60F/40B 24hrs after damage	Phase	+		-		+		-		+		-	
		Peak 1	Peak 2	Peak 1	Peak 2	Peak 1	Peak 2	Peak 1	Peak 2	Peak 1	Peak 2	Peak 1	Peak 2
	Chemical shifts	(-72.66, -74.38)	(-72.82, -74.84)	(-72.85, -73.69)	(-73.03, -74.23)	(-73.06, -73.06)	(-73.23, -73.62)	(-73.26, -72.45)	(-73.41, -72.98)	(-73.44, -71.82)	(-73.63, -72.31)	(-73.66, -71.18)	(-73.82, -71.57)
	# of contours	3		5		6		6		4		1	

Table S2 TFEMA/nBA feed (f) and actual (F) molar ratios in p(TFEMA/nBA) copolymers.

TFEMA/nBA molar feed ratio (f)	Actual p(TFEMA/nBA) molar ratio (F)*
26/74	30/70
35/65	39/61
40/60	45/55
45/55	50/50
50/50	56/44
54/46	59/41
66/34	70/30

*Determined by ^{1}H NMR spectroscopy; % conversion rates after polymerization are 95-98%. The f and F trends are in agreement with the literature.[6]

Table S3 Junction densities (v_j), stored entropy (ΔS_s), and mol. wt. (M_j) as a function of TFEMA/nBA monomer molar ratios in p(TFEMA/nBA) copolymers.

Monomer ratio* (TFEMA/nBA)	v_j (mol/m^3)	ΔS_s (kJ/m^3)	M_j (g/mol)
40/60	31.3	6.6	33710
45/55	36.1	7.0	32941
50/50	39.1	10.8	28813
56/44	34.8	10.3	32010
60/40	30.7	6.6	36878

* Determined by ^{1}H NMR

Table S4 Number averaged molecular weight (M_n), weight averaged molecular weight (M_w), and dispersity (Đ) of p(TFEMA/nBA) with the molar ratios in the 39/61 to 59/41 range.

p(TFEMA/nBA) molar ratio (F)	M_n (g/mol)	M_w (g/mol)	Đ
39/61	45355	127584	2.81
45/55	46318	128764	2.78
50/50	48952	149206	3.04
56/44	49242	142063	2.89
59/41	50038	146311	2.92

Video S1

Self-healing of p(TFEMA/nBA) copolymer films recorded under optical microscope (20X); TFEMA/nBA molar ratio = 50/50. M_n = ~58 kD; Self-healing within 48 hrs under ambient conditions; RH = 40-50%; T=25 ºC; Copolymer synthesis and film formation are described in the Experimental Section. Film dimensions: 1.0 cm x 1.0 cm x 0.45 mm (L x W x T). Cross cuts were made by hand using a razor blade; an approximate cut size: width ~50 μm; depth: ~60-70 μm.

References

1 Davydovich, D. & Urban, M. W. Water accelerated self-healing of hydrophobic copolymers. *Nature communications* **11**, 1-7 (2020).

2 Passaglia, E., Aglietto, M., Ciardelli, F. & Mendez, B. 13 C NMR Characterization of Polymers from 2, 2, 2-Trifluoroethyl Methacrylate. *Polymer journal* **26**, 1118-1123 (1994).

3 Kadimi, A. *et al.* Preparation and dielectric properties of poly (acrylonitrile-co-2, 2, 2-trifluoroethyl methacrylate) materials via radical emulsion copolymerization. *Polymer Chemistry* **10**, 5507-5521 (2019).

4 Flory, P. J. *Principles of polymer chemistry*. (Cornell University Press, 1953).

5 Hornat, C. C., Yang, Y. & Urban, M. W. Quantitative predictions of shape - memory effects in polymers. *Advanced Materials* **29**, 1603334 (2017).

6 Guyot, B., Boutevin, B. & Améduri, B. Etude de la copolymérisation de monomères acryliques fluorés avec le méthacrylate de morpholinoéthyle. *Macromolecular Chemistry and Physics* **197**, 937-952 (1996).
